# A Novel 1,3-Β-Glucanase Gene from the Metagenomic Expression Library of Achatina Fulica’s Digestive Gland

**DOI:** 10.22037/ijpr.2020.1101172

**Published:** 2020

**Authors:** Maris Kurniawati, Purkan, Sri Sumarsih, Afaf Baktir

**Affiliations:** a *Department of Chemistry, Faculty of Science and Technology, Airlangga University, Surabaya, Indonesia. *; b *Faculty of Science and Technology, Universitas Kanjuruhan, Malang, Indonesia.*

**Keywords:** Novel 1, 3-β-glucanase, Achatina fulica, Digestive gland, Metagenomic, Expression library

## Abstract

1,3-β-glucanase enzyme has been proved as antibiofilm by hydrolyzing the main component of extracellular matrix of *C. albicans* polymicrobial biofilm, to prevent resistancy during the use of antibiotics. The aim of this study is to construct a metagenomic expression library from *Achatina fulica*’s digestive gland and to screen for a novel 1,3-β-glucanase genes by using its specific substrate of laminarin. A cDNA expression library was constructed using the λTriplEx2 vector in the *E. coli* strain XL1-Blue. Cre-recombinase circularization was used to convert λTriplEx2 to pTriplEx2 in the *E. coli* strain BM 25.8;then IPTG induction was used to express 1,3-β-glucanase. High-efficiency cDNA library of *A. fulica’*s digestive gland was constructed, from where we obtained seventeen *halo* positive plaques, among them is a novel 1,3-β-glucanase gene designated *MkafGlu1*. Its nucleotide sequence has similarities to the endo-1,3-β-glucanase from *Gossypium hirsutum*, as well as the β-glucanases from *Paenibacillus mucilaginosus*, *Verticillium alfalfa*, and *Cryptopygus antarcticus* of 45%, 40%, 38% and 37%, respectively. An open reading frame of 717 bp encoded a protein of 239 amino acids. A novel 1,3-β-glucanase gene called *MkafGlu1* was successfully expressed in *E.coli* BM 25.8 with activity of 1.07 U mL^-1^.

## Introduction

Group of the β-glycosidases or β-glycoside hydrolases have been used in a variety of applications, including as antibiofilm agents (1, 2, 3), nutritional supplements in feed industries (4, 5), to convert plant biomass into bioenergy (6), fiber modification in pulp and paper industries (7, 8), cotton softening in textile industries, and antideposition in detergent industries (9, 10). An example of β-glycoside hydrolases is 1,3-1,4-β-glucanase, which is used to increase the nutritional value of feed. Barley is the base material of most poultry feed, and it has components of 1,4-β-glucan and 1,3-β-glucan. Both 1,3-1,4-β-glucanase and 1,4-β-glucanase enzymes are used as supplements to hydrolyze β-glucans in poultry feed (11). 

Glycoside hydrolases (GH) are produced by bacteria, fungi and are also found in animals such as mollusks, termites, sea urchins, blue mussels, *Ciona intestinalis,* and *Corbicula japonica *(11). *A. fulica* is a species of mollusk that lives in the tropics and subtropics. This organism feeds on plants as a source of nutrients. GH is stored in its digestive glands, which include 1,6-β-glucanase, 1,4-β-glucanhydrolase, endo-1,4-β-glucanase, chitinase, xylanase, cellulase, lichenase, inulase, hemicellulase, amylase, maltase, and sucrase enzymes. These enzymes play an important role in digesting food sources. The chitinase enzyme hydrolyzes chitin, while the 1,3-β-glucanase enzyme hydrolyzes 1,3-β-glucan (12).

*A fulica* shows extraordinary ability to hydrolyze cellulose and hemicellulose in plant materials. This hydrolizing activity come from microbia live in its digestive system, which is a potential source of antibiofilm enzymes (12, 13). Bacterial and fungal biofilm was a significant medical challenge because extracellular matrix biofilm provide a barrier protective life for bacteria and fungal. The component of biofilm matrix can function to impair antibiotic penetration (1). Hydrolytic enzymes isolated from the *A. fulica *digestive gland hydrolyze the polymeric extracellular matrix of fungal biofilms, especially *Candida albicans*. This enzyme consortium is being developed as an antifungal drug to eradicate *Candida* biofilms for all types of candidiasis pathology. These enzymatic antifungals penetrate *Candida* biofilms, providing an alternative treatment for almost all types of antifungal or multidrug resistant infections. The most promising of these hydrolytic enzymes is a β-glucanase that hydrolyzes the main component of the extracellular matrix of *C. albicans* biofilm. This discovery paves the way for using enzymes from *A. fulica* together with other antifungal compounds to overcome candidiasis infection (13).

Consequently, it is important to explore the digestive gland of *A. fulica* for finding novel potential antibiofilm glycoside hydrolases by metagenomic approach. The screening of gene pools or gene libraries can be pursued through metagenomic techniques, which are divided into two approaches, function-based and sequence-based (14). The function- or activity-based approach uses the expressed activity of cloned sequences to search new genes with targeted function of the genes, without knowing the type of organism from which the sequence originates (15). The sequence-based approach uses to analyze species diversity and make functional associations (16). 

Metagenomic techniques have been used to explore novel genes from various environments and ecosystems (17). Traditional microbiology, microbial genome sequencing and genomics rely upon cultivated clonal cultures, where only 0.1% of environmental microorganism growth on culture medium was made in laboratory. It is different from metagenomic, which is a study of genetic material recovered directly from environmental samples without growing it first. As it does not depend on laboratory cell culture, it is able to recovere all genes in the environment and obtain novel genes. Functional-based metagenomics have the advantage of not requiring the cultivation of microorganisms and without requiring information on existing gene sequences (15). The functional metagenomic was an effective method for finding proteins with true activity, because the selected candidates which expected activity of the interest genes can be expressed properly (18).

Microbiota of the digestive gland of *A. fulica* has identified the dominant bacterial phylum as Proteobacteria, followed by Bacteriodetes and Firmicutes; virus, fungi and archaea are also present (19). There are prokaryotes and eukaryotes in the microbiota, and it is estimated more than 99 % of the microbiota population is unculturable (20). Finding for a gene encoding glycoside hydrolase from the microbiota of the *A. fulica* digestive gland was pursued through screening of the metagenomic library. This study constructed and screened metagenomic library using a laminarin as β-glucan substrate (21) and the λTriplEx2 vector in *E. coli*. 

In the present study, a metagenomic library was constructed using cDNA resulted from Reverse Transcription-Polymerase Chain Reaction (RT-PCR) total RNA from digestive gland *A. fulica*. A gene encoding a novel 1,3-β-glucanase, *MkafGlu1*, was identified through the functional-based approach screening of a phagemid lambda metagenomic expression library of digestive gland *A. fulica*.

## Experimental

**Figure 1:**
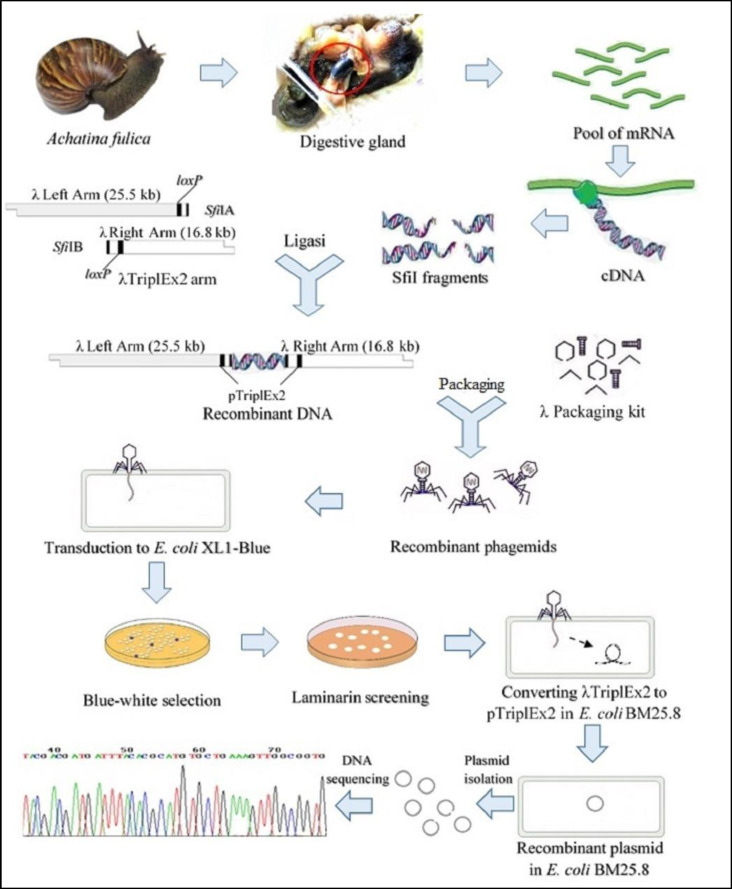
The whole strategy of obtaining novel β-glucanase genes by function-based metagenomic library method

Scheme of this research was carried out by modified from Handelsman J method (22) which slight modification are shown in Figure 1 The salivary glands, crop and esophagus were removed from *A. fulica* and crushed by periodically adding liquid nitrogen, and obtained homogenate RNA isolation.

*Collection of samples and RNA isolation*

Samples of *A. fulica* were obtained from the wild land in Blitar, East Java, Indonesia. The snails were quarantined and fed mustard for three days; then they were anesthetized using chloroform, and part of the intestines were removed. The salivary glands, crop and esophagus were crushed with a mortar in cool conditions by periodically adding liquid nitrogen. RNA isolation was carried out according to the PureZol^TM^ (Bio-Rad, US) instruction manual. Analysis of RNA purity was evaluated by an Optical Density (OD) measurements at 230 nm, 260 nm, and 280 nm using Nanodrop 2000 Spectrophotometer (Thermo Scientific, US). The purity was predicted based on the comparisons of OD_260_/OD_230_ and OD_260_/OD_280_.

*Synthesis of cDNA*

Reverse Transcription - Polymerase Chain Reaction (RT-PCR) using Long Distance-Polymerase Chain Reaction (LD-PCR) method were used to produce double strand cDNA after the first strand cDNA synthesis according to the protocol of the SMART^TM ^cDNA library construction kit (BD Bioscience, US). Amplifications were performed according to the following program: pre-denaturation at 60 °C for two minutes; denaturation at 45–60 °C for 15 min and at 94 °C for two minutes; 20 cycles of PCR comprising denaturation at 94 °C for 15 seconds, annealing at 55–65 °C for 30 seconds, and extension at 68–72 °C for 30 seconds; then post-PCR at 68 °C for five minutes. The cDNA samples were digested by Proteinase K to stop the activity of the DNA polymerase. The PCR products were then purified using a Qiaquick PCR purification kit (Qiagen, Germany). The cDNA was digested using the SfiI enzyme. Size fractionation of the cDNA was performed by the Chroma SPIN-400. Sixteen fractions were collected in separate tubes. Three µL of each fraction was taken for electrophoresis in 1.1% agarose / Ethidium Bromide (EtBr) gel together with 0.1 g of a 1 kb DNA marker (N3232V, New England Biolabs, US) at 150 V for three minutes. The peak fraction was determined by visualizing the intensity of the bands under ultraviolet light. The first three fractions containing large cDNA were combined into one Eppendorf tube and ligated to the λTriplEx2 vector for the cDNA library construction.

*Construction of the cDNA library*

The cDNA and the λTriplEx2 vector were ligated in ratios of 3:2, 1:1 and 2:3 (name of samples L1, L2 and L3 respectively). The lambda phage *in-vitro* packaging of each ligation (L1, L2, and L3) produced an unamplified cDNA library in accordance with the protocols for the MaxPlax^TM^ packaging extract (Epicenter, US), and the product was stored at 4 °C. Stock of the *E. coli* strain XL1-Blue cells was prepared using nutrient agar. Single colony isolates were inoculated into Luria Bertani (LB)/MgSO_4_/maltose broth to prepare the *E. coli* XL1-Blue overnight culture (optical density / OD = 2). The culture was centrifuged, and the pellet was resuspended in 7.5 mL MgSO_4_ 10 mM. The L1, L2 and L3 samples were diluted to 1: 1,000,000. From each packaging product, 1 μL from of the diluted phage was removed and added to 200 μL of *E. coli* XL1-Blue, which was then cultured overnight. 

*Screening of the recombinant phagemid harboring the β-Glucanase genes*

After the recombinant phages transfected the *E. coli* XL1-Blue culture, the bacteria were screened for 1,3-β-glucanase activity by Congo red staining procedure (23). Congo red staining procedure was carried out by blending them in 0.7 % (w v^-1^) of soft agarose overlays containing laminarin (Sigma Chemical Co., US) at a concentration of 0.3 % (w v^-1^), 2.5 mM isopropyl-L-D-thiogalactopyranoside (IPTG), and Congo red dye (1 mg mL^-1^) (24). The bacteria were then incubated at 37 °C for 6–22 h. The recombinant phagemids with 1,3-β-glucanase activity created a *halo* around their plaque.

*Conversion of phagemids λTriplEx2-recombinant to plasmid pTriplEx2-recombinant*

**Figure 2:**
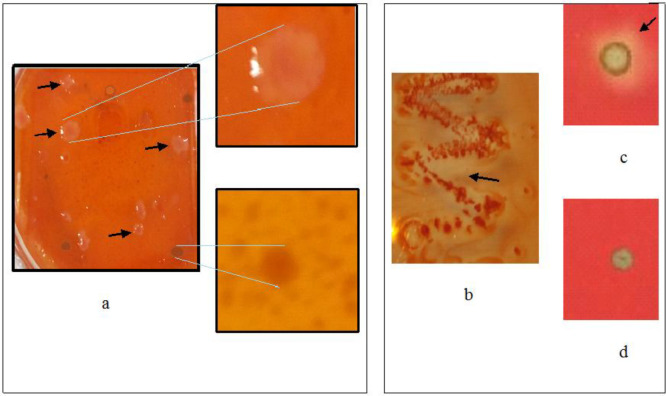
Enzymatic activity assay by a Congo red procedure with laminarine substrate from plaques recombinant harboring the λTriplEx2-MKAFGlu1 (A), clones recombinant harboring the pTriplEx2-MKAFGlu1 (B), cell lysate of recombinant* E. coli* harboring the pTriplEx2-MKAFGlu1 (C), and the control of experiment (D).

The recombinant phagemids with positive *halos* (Figure 2.a) were circularized and converted into plasmid (*pTriplEx2-recombinant*) using the *E. coli* strain BM 25.8 as the host with the method described in the SMART cDNA library construction kit user manual. 

*Isolation and analysis of the pTriplEx2-recombinant*

The recombinant plasmid isolation was performed according to a GeneJET Plasmid Midiprep kit method (Fermentas, Lithuania). The DNA insert was then amplified and sequenced using primers from the SMART^TM^ cDNA library construction kit. Restriction analysis was conducted on the DNA insert using the HindIII enzyme based on the GoTaq Green Master Mix Promega procedure (Promega, US). The treated DNA insert was then electrophoresed at 1.1% agarose/EtBr at a potential difference of 110 V for 30 min. The DNA inserts were sequenced at Macrogen (Seoul, Korea).

*Bioinformatic analysis of the β-glucanase gene*

The nucleotide sequences were aligned using the Clustal W program (25). A phylogenetic tree was generated using MUSCLE (Methodes Algorithmes Bioinformatic Lirmm) (26).

*Expression of the β-glucanase gene*

Clones of the recombinant* E. coli* BM 25.8 were constructed from the recombinant phagemids with positive *halo* on the laminarin agar medium. An inoculum of *E. coli* BM 25.8 was prepared by culturing the bacteria in 15 mL of LB broth medium and shaking at 225 rpm at 31 °C for 12–16 h. Induction with IPTG was performed by adding 15 μL of 100 μM IPTG to the inoculum, which was then incubated while shaking for four hours. An inoculum of 1–2 mL was centrifuged at 5,000 rpm for 20 min. Cell pellets were resuspended in 100 μL of phosphate buffer saline. The cell pellets were disrupted by adding 200 μL of lysis buffer solution and incubated for 20 min at 37 °C. The lysate was centrifuged at 12,000 rpm for ten minutes. The supernatant contained the crude glucanase and was used to perform the glucanase activity assay modified from cellulase assay (27) and enzymatic plate assay (12).

To investigate the biochemical properties of MKAFGlu1, a clone of the gene was expressed in *E. coli* BM 25.8 with IPTG induction. MKAFGlu1 expression was demonstrated using three kinds of glucanase activity assays: the *halo* plate assay of recombinant *E. coli* BM25.8 in LB-laminarine (1 mg mL^-1^) agar culture (Figure 2.b); the cell lysate enzyme assay on wells of laminarine (1 mg mL^-1^) agar (Figure 2.c); and the dinitrosalicylic acid (DNS) method (24) of determining enzyme activity.

## Result and Discussion

*Construction of the metagenomic expression library*

Digestive gland of *A. fulica* is a suitable source of novel enzymes exploration by metagenomic method, especially glycoside hydrolases, as it a herbivorous with great ecological and able to quickly hydrolyse variety of vagetable. These enzymes come from microbiota inside the digestive gland (12, 19). We assumed digestive gland of *A. fulica* is a glycoside hydrolise reservoirs represents a rich and source of novel enzymes. 

Total RNA was isolated from part of the digestive gland of *A. fulica* that included crop and other parts of the intestine, which are reservoirs of glycoside hydrolases (19). Induction of mRNA expression for glycoside hydrolases was carried out by mustard feeding in three days quarantine. 

The total RNA concentration obtained from 100 mg of a digestive gland sample was 2,343.2 ng μL^-1^. The RNA purity test was performed by measuring nanodrop absorbance at wavelengths of OD_260_ and OD_280_ (OD_260_/OD_280_) to indicate the amount of protein contaminants. The RNA purity test also measured the absorbance ratio of OD_260_/OD_230_ to indicate the amount of polysaccharide, phenol, and chaotropic salt contaminants (28). The OD_260_/OD_280_ ratio of the collected RNA was 1.95, falling within the purity criteria of 2.0 + 0.1 (29). The OD_260_/OD_230_ ratio was 1.69, beyond the purity criteria. The contaminating polysaccharides, phenols or salts may have been due to residuals of the guanidine or β-mercaptoetanol buffer used during the RNA isolation process. 

**Figure 3:**
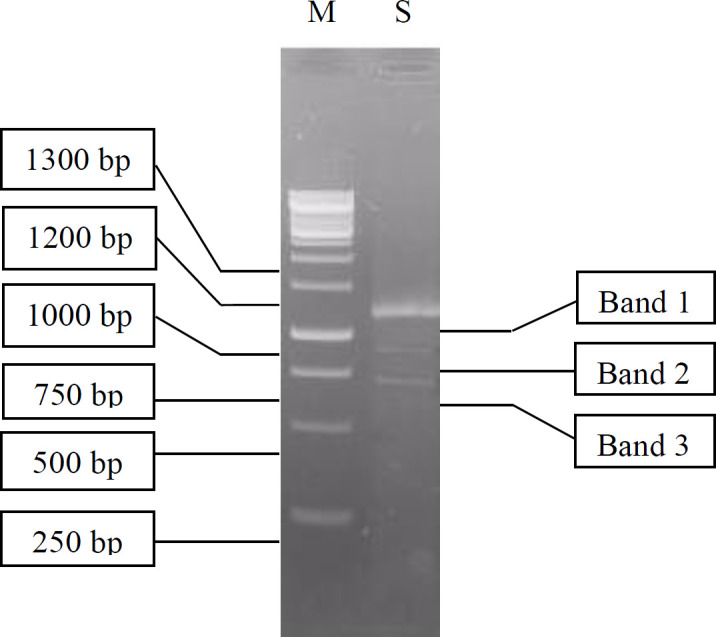
Visualization of cDNA synthesized using RT-PCR (S) on 1.1% agarose gel. M is a 1 kb DNA ladder marker

Isolation and purification of the total RNA extracted from digestive gland of *A. fulica* was an important stage in the construction of the cDNA library (30). The electrophoresis of the total RNA resulted in a smear band between 0.5 kb–2 kb (data not shown) indicating varying sizes of RNA. Electrophoresis of the RT-PCR RNA samples produced a long smear, distributed from 250 bp to 1,300 bp, with one clear band (1,100 bp) and two obscure bands (900 bp and 700 bp) as shown in Figure 3.

The cDNAs smaller than 500 bp were eliminated in the fractionation process to prevent the formation of library with short inserts or non-recombinant clones. The remaining cDNAs were separated into 16 fractions. The cDNA in fractions 8 to 16 had band sizes of 700, 900, and 1100 bp. The three fractions with the largest cDNA size were selected and combined. The largest cDNA size was chosen, because it was assumed not degraded, so the full length of the cDNA sequences was expected to be expressed during the screening process. The next step was to ligate the cDNA into the lambda vector.

The critical factor in obtaining an efficient transformation was the concentration ratio of cDNA to phage in the ligation reaction. The optimal ratio must be determined empirically for each cDNA/phage combination. To construct the best possible metagenomic library, three parallel ligations were set up using the ratios shown in Table 1. The ligation efficiencies are presented in Table 1 for the unamplified libraries.

According to Table 1, the optimal ligation ratio of cDNA to phage was 2:3, which created unamplified library of 2.8 x 10^8 ^pfu mL^-1^. This number is more than the minimum standard of 1.7 x 10^5^ pfu mL^-1 ^(31). While, amplified lambda lysate packaging libraries with the 2:3 ligation ratios, showed plaque titers of 1.1 x 10^10 ^pfu mL^-1^, indicating the successful construction of amplified cDNA libraries from the digestive gland of *A. fulica*. This data was the best compared to the previous reports of metagenomic library constructions. Among them are: 1) Titer of cDNA library from water-stressed plantlets regenerated *in-vitro* of Populus hopeiensis that was reported 1.69 x 10^9^ pfu mL^-1^ (32). 2) Titer of cDNA library from human liver tissue with chronic hepatitis was reported 1.49 x 10^9 ^ pfu mL^-1^ (33). 3). Titer of cDNA metagenomic library from environmental samples was reported 8.00 x 10^9 ^pfu mL^-1^ (34). 4) Titer of cDNA metagenomic library from the endangered hu sheep was reported 1.09 x 10^10 ^pfu mL^-1^ (35).

*Screening and analysis of the recombinant plaques harboring the 1,3-β-glucanase genes*

Screening of the recombinant plaques for genes encoding 1,3-β-glucanase performed using a Congo red staining method on a laminarin substrate, we found 17 plaques. These recombinant plaques that expressed glucanase, especially 1,3-β-glucanase, created a white spot surrounded by a transparent region or *halo* (Figure 2.a). The recombinant plaques could be only detected enzyme activity if the gene was transcribed, translated, and folded correctly in the host cells (27). The success of detecting 1,3-β-Glucanase at the screening stage of the metagenom library depends on the enzyme released from the cell or cell lysis, which then reacts with the laminarin substrate. Each of the 17 plaques was then converted from lambda phages into plasmids with circularisation in *E. coli* BM25.8, so we have 17 recombinant *E. coli* clones harboring different genes. Phage circularization was achieved by transducing recombinant phagemide in the lysogenic host *E. coli* BM25.8. In this recombination stage lysogenic hosts were used because the host does not undergo lysis during the transduction process. This phage circularization step produced recombinant plasmid clones, which were then isolated and analyzed (data not shown).

Moreover, we randomly selected eight clones of recombinant *E. coli* for PCR amplification, i.e. clones number 1, 3, 6, 8, 10, 11, 14, and 17. Clones number 1 and 6 produced the same three bands on electrophoresis, indicating that they contained similar inserts. While, clones 8 and 10 were also predicted to contain the same insert; clones 3 and 11 produced one band with different sizes. PCR colonies against clones number 14 and 17 did not produce any band on electrophoresis. This can be caused by very small levels of amplicon DNA (data not shown).

*Sequencing and analysis of 1,3-β-glucanase gene*

**Figure 4.**
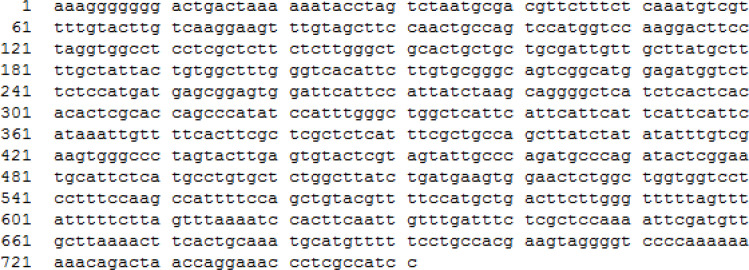
The nucleotide sequence of β-glucanase gene MKAFGlu1

One clone of recombinant *E. coli* 25.8 expressing 1,3-β-glucanase activity was chosen for sequencing analysis. The sequencing primer was a T7 promoter (5’-TAATACGACTCACTATAGGG-3’). The novel sequence of this DNA insert, named *MkafGlu1*, is shown in Figure 4.

*MkafGlu1* is a 751 bp nucleotide, contains an open reading frame (ORF) between base pairs 35 and 751 (encoding 717 bp nucleotide) with 5’ and 3’ untranslated regions of 34 and 1 bp. The Macrogen nucleotide reader can read more than 751 bp. *MkafGlu1* was aligned to nucleotides from the NCBI database using the Clustal W program. The nucleotide sequence of *MkafGlu1* has 45% similarity to the endo-1,3-β-glucanase from *Gossypium hirsutum*, 40% similarity to the β-glucanase from *Paenibacillus mucilaginosus*, 38% similarity to the β-glucanase from *Verticillium alfalfa*, and 37% similarity to the β-glucanase from *Bacillus amyloliquefaciens* and *Cryptopygus antarcticus*. The very low similarity percentages indicate that *MkafGlu1* is a novel 1,3-β-glucanase gene. The nucleotide sequence of *MkafGlu1* has been deposited in GenBank (accession number MH206587).

In this research we revealed a novel protein of 239 amino acids (AA) based on deduction from the *MkafGlu1* open reading frame. The MkafGlu1 protein was predicted having molecular weight of 26,29 kDa. Database homology search of the deduced AA sequences was conducted by using the Basic Local Alignment Search Tool (36) of National Center for Biotechnology Information (NCBI). The deduced AA sequence matched with 1,3-β-glucanase from H. discus hannai (accession code No. AB488493) and *P. sachalinensis* (accession code No. AY308829) with homology 45 and 46%, respectively. According to gene cluster encoding enzymes, MkafGlu1 belongs to glycosyl hydrolase families 16 (GH16). 

**Figure 5:**
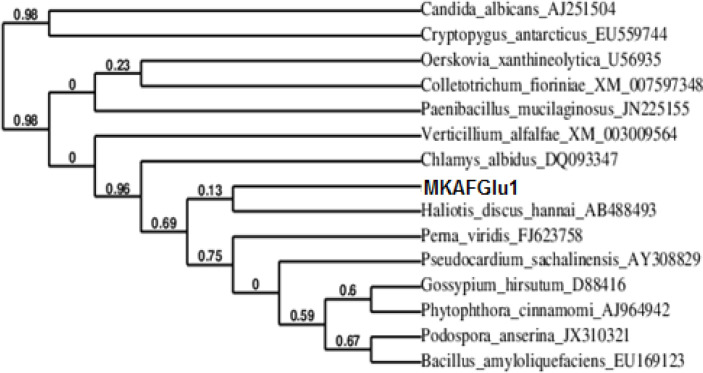
The phylogenetic tree of novel β-glucanase gene MKAFGlu1 with closely related proteins

A phylogenetic tree of the nucleotide sequence was constructed to explore the evolutionary relationship between *MkafGlu1* and the other β-glucanase genes. As shown in Figure 5, *MkafGlu1* is a member of the 1,3-β-glucanase family.

*Expression of 1,3-β-glucanase gene and assay of its enzyme activity*

1,3-β-glucanase activity expressed by plaque and *E.coli* recombinants during metagenomic construction until selection of MKAFGlu1 was showed in Figure 2 As shown in Figure 2.a, plaque harboring the λTriplEx2-MKAFGlu1 was created *halo* on the LB agar that contained laminarin. Meanwhile, Figure 2 b show clones of recombinant *E. coli* harboring the pTriplEx2-MKAFGlu1 which also created *halo* on same LB agar composition. Whereas Figure 2.c show 1,3-β-glucanase activity of the recombinant cell lysate poured into the laminarin agar well, produced a *halo* of β-glucanase activity. 

The enzyme activity of the recombinant *E. coli* BM25.8 cell lysate was also determined quantitatively by the DNS method. It produced 1,3-β-glucanase activity of 1.07 U mL^-1^, where 1 U of 1,3-β-glucanase activity is the amount of enzyme liberating 1 mmol of reducing sugar, calculated as glucose per minute per mL in experimental conditions. The flow diagram of this research is presented in Figure 1.

Finally 1,3-β-glucanase recombinant *MkafGlu1 *was isolated from the recombinant *E. coli* BM25.8 and characterized. The *MkafGlu1* had optimal temperature and pH activity of 40 ^o^C and 7.0, respectively. 

**Table 1:** Ligations process using 3 different rasio of cDNA to phage vector

**No**	**I** **nitials**	**cDNA : ** **phage v** **ector**	**Plaque (pfu/m** **L** **)**
1.	L1	3 : 2	9 x 10^7^
2.	L2	1 : 1	1.5 x 10^8^
3.	L3	2 : 3	2.8 x 10^8^

## Conclusion

A metagenomic expression library of the digestive gland of *A. fulica* containing unamplified library titers of 2.8 x 10^8^ pfu/mL and amplified library titers of 1.1 x 10^10^ pfu/mL have been constructed. Screening of the library against laminarin resulted in 17 *halo* plaques. A novel 1,3-β-glucanase gene, *MkafGlu1*, was successfully cloned from the DNA metagenome of *A. fulica*’s digestive gland*.* The optimal temperature and pH of *MkafGlu1* activity were observed at 40 ^o^C and 7,0, respectively.
